# CircularRNA Hsa_circ_0093335 promotes hepatocellular carcinoma progression via sponging miR‐338‐5p

**DOI:** 10.1111/jcmm.17991

**Published:** 2023-10-14

**Authors:** Xiangyu Qin, Lingyu Zhou, Yaojie Shen, Yuwei Gu, Jia Tang, Junwei Qian, An Cui, Mingquan Chen

**Affiliations:** ^1^ Department of Infectious Diseases, Shanghai Key Laboratory of Infectious Diseases and Biosafety Emergency Response, National Medical Center for Infectious Diseases, Huashan Hospital Fudan University Shanghai China; ^2^ Department of Emergency Medicine, Huashan Hospital Fudan University Shanghai China; ^3^ Department of Rehabilitation Medicine Huashan Hospital Shanghai China; ^4^ Department of Infectious Diseases, Peking Union Medical College Hospital Chinese Academy of Medical Sciences Beijing China

**Keywords:** hepatocellular carcinoma, Hsa_circ_0093335, miR‐338‐5p, treatment

## Abstract

Circular RNAs play an important role in the development of various malignancies, including hepatocellular carcinoma (HCC). Nevertheless, the role of Hsa_circ_0093335 (circ0093335) in HCC has not yet been explored. To investigate the biological effects and molecular mechanisms of circ0093335 on HCC. Circ0093335 expression was detected in HCC cells and clinical specimens using qRT‐PCR. The association between circ0093335 expression and HCC patients' clinical characteristics was determined using SPSS. The role of circ0093335 in HCC was estimated by overexpression and knockdown experiments in vitro and in vivo. qRT‐PCR, nucleoplasma separation assay, FISH assay, RIP, dual luciferase reporter assay and rescue assay were used to validate the regulatory effect of circ0093335 on miR‐338‐5p. The study findings showed that circ0093335 was upregulated in HCC. High circ0093335 expression was linked with the tumour‐node‐metastasis stage and microvascular tumour invasion. circ0093335 is greatly involved in HCC cell proliferation, aggressive ability and mouse tumour growth, according to many in vitro and in vivo tests. Mechanistically, circ0093335 downregulated miR‐338‐5p expression by sponging, consequently promoting HCC progression. Our research indicated that circ0093335 might be a target for HCC therapy since it promotes tumour progression by acting as a miR‐338‐5p ‘sponge’.

## 1 | INTRODUCTION

Hepatocellular carcinoma (HCC), the most frequent primary liver cancer, is the third cause of cancer‐related death around the world.^1^ Chronic hepatitis virus infection (HBV/HCV), alcohol abuse and non‐alcoholic fatty liver disease, are crucial risk factors for HCC.^2^, ^3^, ^4^ Most patients with HCC in clinical practice have advanced disease when they are finally diagnosed due to the unclear symptoms in the early stages and the constraints of employing ultrasound every 6 months (with or without alpha‐fetoprotein), most HCC patients in clinical practice have advanced disease when they are finally diagnosed.^5^, ^6^ The outcomes of patients with HCC remain unsatisfactory despite substantial advancements in HCC management in the past 10 years, with treatment regimens tailored to patients' tumour stages and projected benefits of major interventions.^2^, ^7^ Further research into the molecular elements and their signalling pathways that influence the emergence and development of HCC malignancies is needed because they may represent a therapeutic target for the disease.

Non‐coding RNAs (ncRNAs) are considered key players in cancer biology and participate in gene regulation. When ncRNAs are dysregulated, they contribute to the development of cancer.^8^ Circular RNAs (circRNAs), a novel family of ncRNAs produced by back‐splicing pre‐mRNAs into stable closed‐loop architecture without 5′ caps and 3′ tails, are implicated in biological processes that contribute to tumorigenesis.^9^, ^10^ Most circRNAs are mostly found in the cytoplasm and can take part in gene regulation through sponging miRNAs to stop them from interacting with target mRNAs.^11^, ^12^, ^13^ Recent developments in research have indicated that circRNAs are dysregulated in HCC and are involved in the emergence of HCC. Moreover, some circRNAs can act as biomarkers for therapeutic targets or clinical diagnosis.^14^, ^15^, ^16^ However, further studies are needed to fully understand how circRNAs act in HCC.

BMI1, a proto‐oncogene, regulates a number of physiological processes, including cell differentiation and self‐renewal, and proper gene silencing, all of which are crucial for biological function.^17^, ^18^ A growing body of evidence points to BMI1 as a crucial gene in carcinogenesis. It has been reported to contribute to the development of tumours, including multiple myeloma, and breast and gastric carcinomas.^19^, ^20^, ^21^ BMI1 is highly expressed and greatly contributes to the carcinogenesis and development of HCC, just like in other malignancies.^22^, ^23^, ^24^ Consequently, we hypothesized that circRNAs derived from BMI1 are involved in the development of HCC. Hsa_circ_0093335 (circ0093335), reported in oesophageal cancer, was formed by back‐splicing of exons 6–12 of BMI1 and had an entire length of 589 bp.^25^ We speculated that circ0093335 is involved in the progression of HCC and should be investigated more.

In this research, we identified and confirmed circ0093335 existence in HCC cell lines and its expression in HCC clinical specimens. We further studied the relationships between circ0093335 expression and clinical parameters. Moreover, we showed that circ0093335 promotes HCC by sponging miR‐338‐5p, suggesting a possible HCC therapeutic target.

## 2 | MATERIALS AND METHODS

### 2.1 | Transfection and cell culture

Human liver cell (LO2), HCC cells (Huh7, Hep3B, MHCC97H and HCCLM3) and HEK293 cell, which was bought from the Shanghai Chinese Academy of Sciences cell bank for use in this work. At 37°C, 5% CO2, cells were grown in the appropriate medium with penicillin G, streptomycin, and fetal bovine serum (Sigma Aldrich). Sangon Biotech provided the pcDNA3.1‐circ0093335, sh‐circ0093335, miR‐338‐5p mimic and negative controls (NCs) for cell transfection. Transfection was conducted with Lipofectamine^TM^ 3000 (Invitrogen) under the kit manuals.

Sequence of sh‐circ0093335: CCTAATACTTTCCAGGATTTTTTCAAGAGAAAAATCCTGGAAAGTATTAGGTTTTTT.

### 2.2 | Patient samples

Clinical specimens were collected from 71 HCC patients at Huashan Hospital from 2013 to 2017. For the target gene expression test, these samples were stored at −80°C. All patients' clinicopathological data was gathered. The Huashan Hospital's ethical committee issued the study the stamp of approval. All patients who had signed up provided their informed consent and agreement.

### 2.3 | RNA extraction and quantitative real‐time polymerase chain reaction assay(qRT‐PCR)

TRIzol Regent was used for total RNA extraction of patient samples and cell lines (Invitrogen). RNA quality was tested by NanoDrop 2000 (Thermo Scientific). FastQuant RT Kit or miRNA RT Kit was used for reverse transcription (TIANGEN). Super‐Real PreMix Plus Kit or the miRNA qPCR Detection Kit (TIANGEN) was used to analyse target gene expression in Roche LightCycler480. GAPDH expression and U6 expression were employed as internal control, respectively. The 2−∆∆Ct method was served for statistical analysis.

Primer sequences lists:

1. circ0093335‐Convergent‐F: ATGGACATACCTAATACTTTCCAGGAT.
2. circ0093335‐Convergent‐R: TCCAGGTAACGAACAATACACGT.
3. circ0093335‐Divergent‐F: ATGGACATACCTAATACTTTCCAGGAT.
4. circ0093335‐Divergent‐R: TCCAGGTAACGAACAATACACGT.
5. GAPDH‐F: ATTGTACAGCCCGTCCCCAA.
6. GAPDH‐R: GAGTCGGCTAGGTGCG.
7. miR‐338‐5p‐F: GTTCACCACCTTCTCCAC.
8. U6‐F: CTCGCTTCGGCAGCACA.

### 2.4 | RNase R treatment assay

RNA was mixed with RNase R Kit (Beyotime) and incubated (15 min, 37°C) for the RNase R treatment experiments. To measure the levels of RNA expression, qRT‐PCR was used.

### 2.5 | Cell counting kit‐8 (CCK‐8) assay and transwell assays

Cells were seeded at the proper concentrations into 96‐well plates. Every well‐received CCK‐8 reagent addition at 0, 24, 48 and 72 h, respectively, was incubated for 1 h. Subsequently, using the MRX II absorbance reader (Dynex Technologies), the absorbance (450 nm) of each well was measured. The transwell experiments examined the capacity for cell invasion and migration. Following the addition of 200 μL of cells (adjusted to 2.5 × 10^5^ cells/mL) to the transwell upper chamber (Corning) with or without matrix (Corning) and 600 μL medium (10% FBS) to the lower room. The upper was swabbed after 24–48 h of incubation. The invaded and migrating cells surface were fixed (4% polyformaldehyde) and stained (0.1% crystal violet) for 15 min, respectively. With the use of an inverted microscope (Olympus), pictures were captured.

### 2.6 | Plate clone formation assay

The 6‐well plate (Corning) was seeded with 400 cells, and the cells were spread equally by gently shaking the plate. Every 2–3 days, cell growth was observed with an Olympus microscope (Japan), and the media was changed often. The cells were fixed (4% polyformaldehyde), stained (0.1% crystal violet) (Beyotime) and photographed 10–14 days later.

### 2.7 | Xenograft tumour model

BALB/c female nude mice, which are 4–6 week old, were prepared at Shanghai Model Organisms Center. Each nude mouse received a cell injection (1 × 10^7^/mouse) into the right armpit. Mice were euthanized at 35 days. The transplanted tumours were taken out, photographed and the tumour volume (TV) was assessed every 7 days.

### 2.8 | Haematoxylin‐eosin (H&E) and immunohistochemical (IHC) staining

Mouse tumours were fixed in paraffin and divided into 5 μm slices. 4% paraformaldehyde was used to fix the tumour tissues of mice, which were then ethanol‐dried, paraffin‐embedded, cut into 4 μm slices and treated with 3% H_2_O_2_ solution. After being treated with a blocking solution for 15 min, the slices were incubated (4°C) overnight with antibodies. The recovery processes followed the pattern already mentioned. A microscope was used to take pictures (Olympus). Double‐blind reviewers assessed the staining outcomes.

### 2.9 | Nuclear and cytoplasmic extraction and fluorescence in situ hybridization (FISH)

An RNA Sub‐cellular Isolation Kit was used to separate the RNA into its nuclear and cytoplasmic portions (Active Motif). To gauge the levels of RNA expression, qRT‐PCR was used.

Sangon Biotech created FAM‐labelled probes (5‐FAM‐CACUCAGCACCAGGAUAUUGUU‐3) and cy3‐labelled probes (5‐CY3‐AUCCUGGAAAGUAUUAGGUAUGUCC‐3) to detect miR‐338‐5p and circ0093335 respectively for FISH experiments. Cells were fixed, added pre‐hybridization buffer and hybridized overnight at 37°C. Nucleus staining regent is 4′,6‐diamidino‐2‐phenylindole. Under an orthomorphic fluorescent microscope, the images were seen and recorded.

### 2.10 | RNA immunoprecipitation (RIP) assay

RIP kit (Merck) was used to conduct the RIP assay. After being lysed with RIP lysis solution, cells were treated with magnetic beads (4°C, 6 h) preconjugated with either an anti‐AGO2 antibody or anti‐IgG antibody. After that, proteinase K was used to wash and digest the magnetic beads. To analyse pure RNA, qRT‐PCR was used.

### 2.11 | Dual‐luciferase reporter gene assays

The circ0093335 gene was cloned and inserted into the psiCHECK‐2 vector along with the appropriate mutations of the miR‐338‐5p. HEK293 were transfected with luciferase reporter plasmids of NC, miR‐338‐5p mimics, wild‐type vector (WT), or mutant vector (MUT) in 6‐well plates using Lipofectamine3000. The Luciferase Reporter kit was applied to determine luciferase activity (Promega).

### 2.12 | Statistical analysis

Using SPSS Inc., results are shown as mean ± SD. Graph Pad Prism7 was applied to create the graphs. The statistic was determined by the one‐way analysis of variance (anova) test or the Student's *t*‐test (paired, two‐tailed).

The following symbols signify *p*‐values: *p < 0.05, **p < 0.01 and ***p < 0.001. Statistics were seen as significant at ‘*p* < 0.05’.

## 3 | RESULTS

### 3.1 | circ0093335 is highly expressed in HCC

**FIGURE 1:**
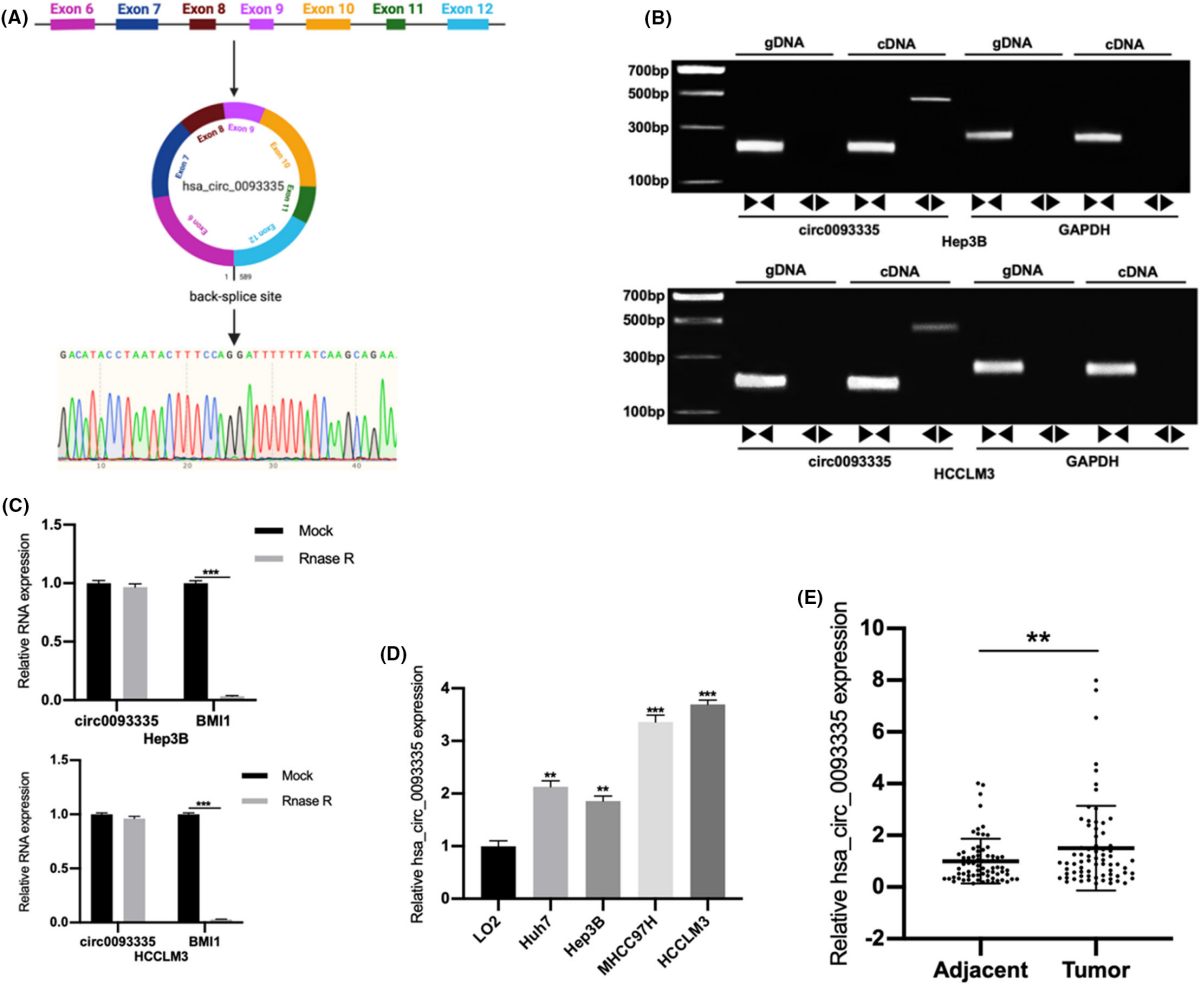
Identification and characteristics of circ0093335 in HCC. (A) Diagram showing the creation of circ0093335 and Sanger sequencing outcomes. (B) Divergent primers detected circ0093335 in cDNA. GAPDH detection was a control. ►◄convergent primers, ◄►divergent primers. (C) RNase *R* tests to verify circ0093335 stability. (D) Circ0093335 expression in LO2 and HCC cells. (E) Circ0093335 expression in 71 HCC specimens. *p<0.05, **p<0.01, and ***p<0.001.

The junction site of circ0093335 (derived from chromosome 10: 22615359–22,617,627) was verified using Sanger sequencing. It consists of seven consecutive exons in the BMI1 gene (Figure 1A). cDNA and gDNA templates were taken from Hep3B and HCCLM3 cells for convergent and divergent primers designed to characterize the ring structure. The PCR results were assessed by agarose gel electrophoresis. The outcomes revealed that only cDNA, not gDNA, was amplified by divergent primers for circ0093335 (Figure 1B). Moreover, the RNase R digestion experiments revealed that circ0093335 was more stable than BMI1 (Figure 1C).

Circ0093335 expression was tested in both HCC cells and LO2. The findings showed a considerable upregulation of circ0093335 expression in HCC cells. The expression level was relatively high in HCCLM3 cells and relatively low in Hep3B cells (Figure 1D). Circ0093335 expression levels in 71 HCC specimens were ascertained using qRT‐PCR. The findings revealed that circ0093335 was significantly upregulated in the tumour (Figure 1E). Based on the medium expression, patients were categorized into two groups. The relationships between clinicopathological variables and circ0093335 expression levels were evaluated. The findings revealed that higher expression of circ0093335 correlated with advanced tumour‐node‐metastasis (TNM) stage and tumour microvascular invasion (MVI) (*p* < 0.05; Table 1). However, no statistically significant differences in age, gender, tumour size, or hepatitis B virus infection were observed. These findings indicate that circ0093335 is elevated in HCC cell lines and tissues and plays an important role in HCC oncogenesis.

**TABLE 1:** Association between clinical features and circ0093335 expression of hepatocellular carcinoma patients.

Clinical features	Circ0093335 expression		*p*‐value
	Low expression (≤median)	High expression (>median)	
Number	37	34	
Age(years)			0.959
≤50	10	9	
>50	27	25	
Gender			0.176
Male	28	30	
Female	9	4	
Tumour size(cm)			0.211
≤5	9	13	
>5	28	21	
Microvascular invasion			0.034*
Yes	30	32	
No	7	2	
TNM stage			0.032*
I + II	19	9	
III + IV	18	25	
HBV infection			0.174
Yes	31	32	
No	6	2	

* *p* < 0.05.

### 3.2 | Circ0093335 promoted HCC progression in vitro and in vivo

**FIGURE 2:**
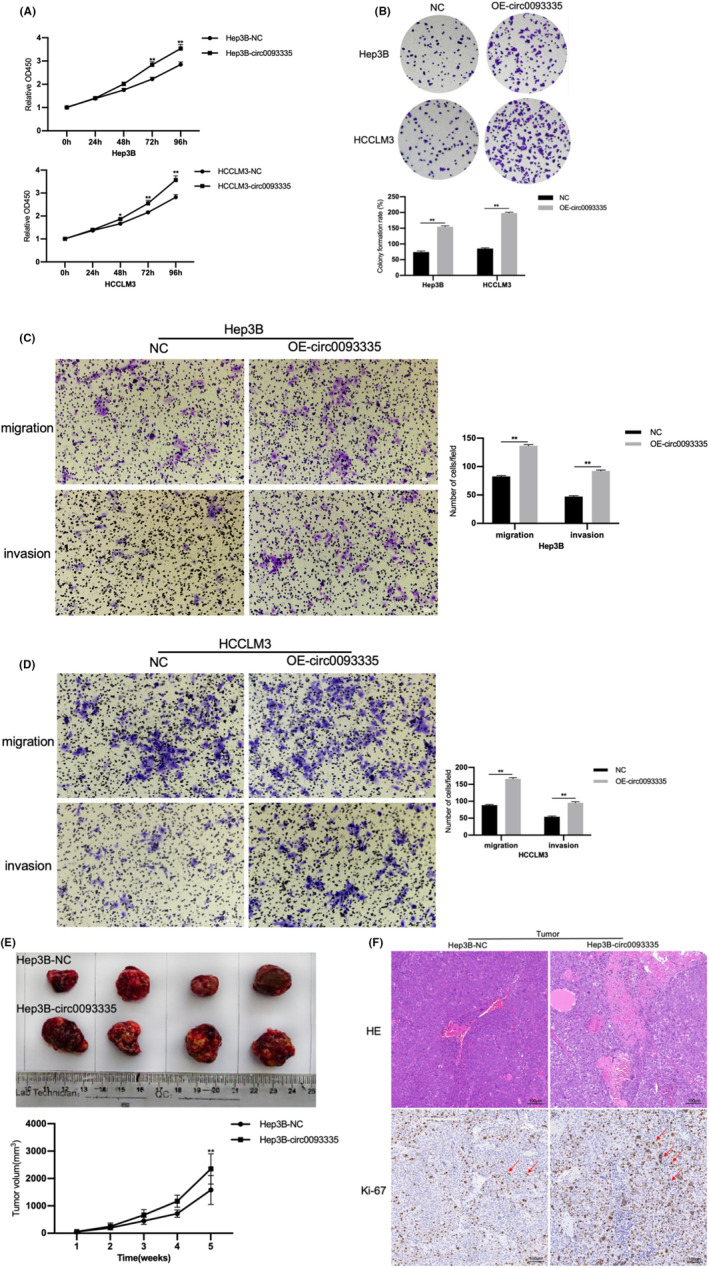
Circ0093335 overexpression enhanced HCC growth. (A) The proliferation of NC or OE‐circ0093335 cells was tested by CCK‐8 regent. (B) The colony‐forming ability of NC or OE‐circ0093335 cells was determined. (C, D) Transwell assays with or without matrix was used to assess the aggressive ability of NC or OE‐circ0093335 cells, scale bar and 50 μm. (E) Tumours were harvested on Day 35 after subcutaneous injection (1 × 10^7^ cells, *n* = 4). Tumour volume (TV) was recorded every 7 days for the first 35 days following the initial injection. (F) Representative images of HE staining was carried out in two groups (Hep3B‐NC and Hep3B‐circ0093335), and the IHC test was used to identify Ki‐67 expression, scale bar, 100 μm. *p < 0.05, **p < 0.01, and ***p < 0.001.

The impact of circ0093335 on HCC was investigated in vitro using CCK‐8 and colony‐forming experiments. The outcomes revealed that circ0093335 overexpression markedly facilitated the growth of Hep3B and HCCLM3 (Figure 2A, B). Additionally, the impact of circ0093335 on the mobility and aggression ability of cells was investigated using transwell experiments. The outcomes revealed that circ0093335 overexpression significantly promoted Hep3B and HCCLM3 to migrate and invade (Figure 2C, D). These results prove that circ0093335 increases HCC cell proliferation and motility.

Hep3B cells transfected with NC lentiviral vectors (Hep3B‐NC) or circ0093335‐overexpressed lentiviral vectors (Hep3B‐circ0093335) were injected subcutaneously to investigate the impact of circ0093335 on mouse tumour growth in vivo. The TV was markedly increased in the Hep3B‐circ0093335 group compared with the Hep3B‐NC group (Figure 2E). HE staining of the tumour in each group revealed that the Hep3B‐circ0093335 group had more tumour features than the Hep3B‐NC group (Figure 2F). Furthermore, the immunohistochemistry assay detected the expression of the proliferative indicator Ki‐67 in each group. Ki‐67 expression was relatively high in the Hep3B‐circ0093335 group (Figure 2F). These results prove that circ0093335 promotes HCC tumorigenesis in vivo.

### 3.3 | Knockdown of Circ0093335 inhibits HCC progression in vitro and in vivo

**FIGURE 3:**
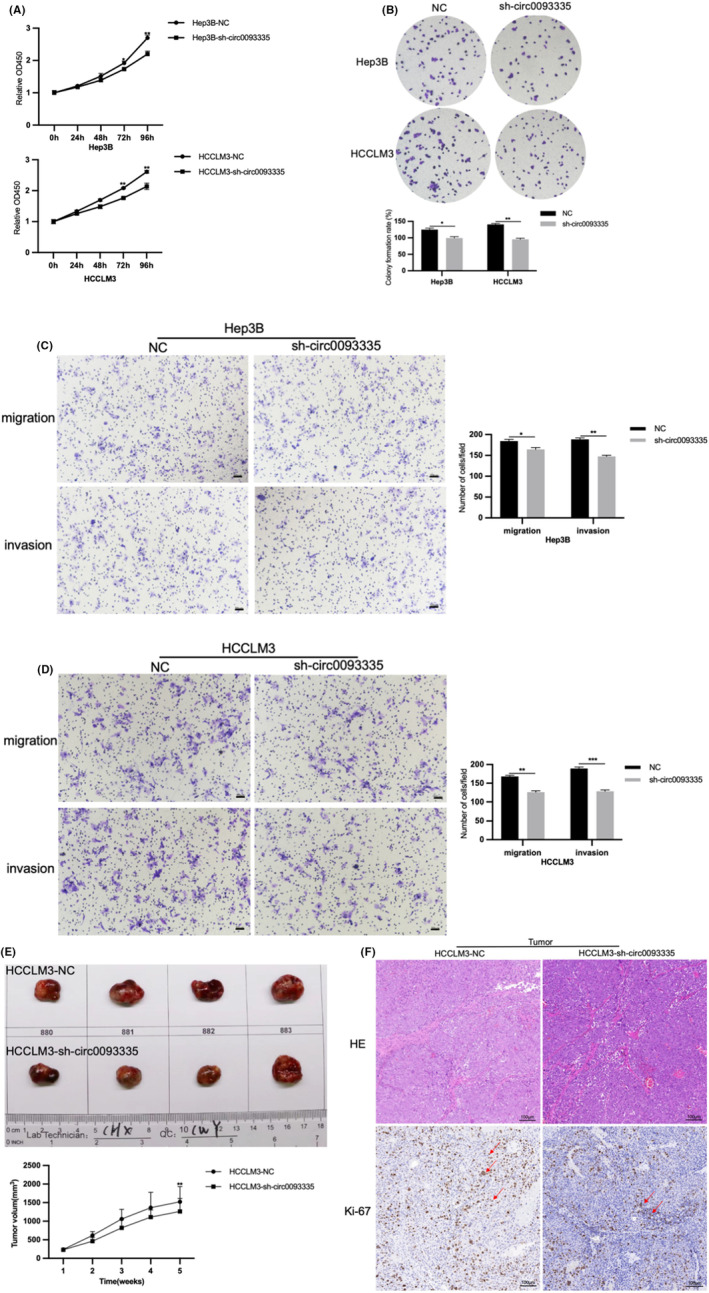
Knockdown of circ0093335 inhibits HCC progression in vitro and in vivo. (A) The proliferation of negative control (NC) or sh‐circ0093335 cells was tested by CCK‐8 regent. (B) The colony‐forming ability of NC or sh‐circ0093335 cells was determined. (C,D) Transwell assays with or without matrix was used to assess the aggressive ability of NC or sh ‐circ0093335 cells, scale bar, 50 μm. (E) Tumours were harvested on Day 35 after subcutaneous injection (1 × 10^7^ cells, *n* = 4). TV was recorded every 7 days for the first 35 days following the initial injection. (F) Representative images of HE staining was carried out in two groups (HCCLM3‐NC and HCCLM3‐sh‐circ0093335), and the IHC test was used to identify Ki‐67 expression, scale bar, 100 μm. *p < 0.05, **p < 0.01, and ***p < 0.001.

The impact of knockdown of circ0093335 on HCC was investigated in vitro using CCK‐8 and colony‐forming experiments. The outcomes revealed that knockdown of circ0093335 markedly inhibited the growth of Hep3B and HCCLM3 (Figure 3A,B). Additionally, the impact of knockdown of circ0093335 on the mobility and aggression ability of cells was investigated using transwell experiments. Knockdown of Circ0093335 significantly inhibited Hep3B and HCCLM3 to migrate and invade, according to the findings (Figure 3C,D). These results prove that knockdown of circ0093335 inhibited HCC cell proliferation and motility.

HCCLM3 cells transfected with NC lentiviral vectors (HCCLM3‐NC) or sh‐circ0093335 lentiviral vectors (HCCLM3‐sh‐circ0093335) were injected subcutaneously to investigate knockdown of circ0093335 impact on mice tumour growth in vivo. Compared to the Hep3B‐NC group, the TV was decreased in the HCCLM3‐sh‐circ0093335 group (Figure 3E). HE staining of the tumour in each group revealed that the HCCLM3‐NC group had more tumour features than the HCCLM3‐sh‐circ0093335 group (Figure 3F). Furthermore, the IHC assay detected the expression of the proliferative indicator Ki‐67 in each group. The Ki‐67 expression was relatively high in the HCCLM3‐NC group (Figure 3F). Knockdown of circ0093335 inhibited HCC tumorigenesis in vivo, according to these findings.

### 3.4 | Circ0093335 functions as a sponge of miR‐338‐5p

**FIGURE 4:**
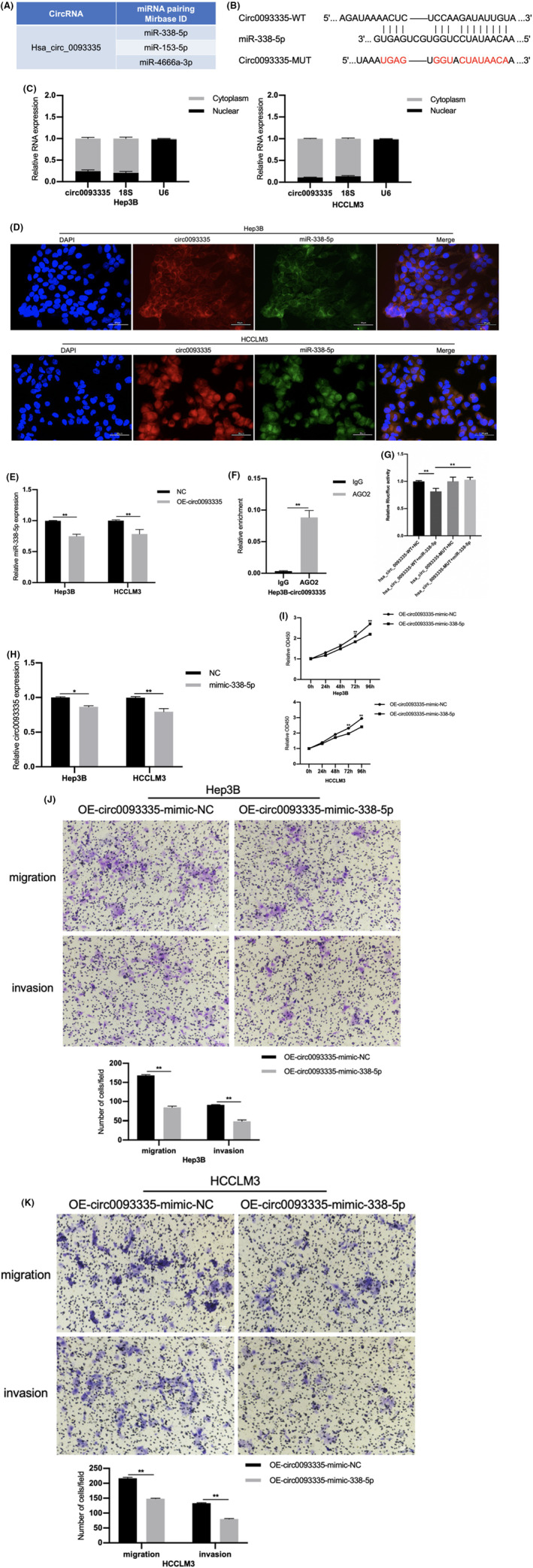
miR‐338‐5p is the target of circ0093335. (A) Prediction of potential miRNA targets for circ0093335 by Circinteractome. (B) Schematic representation of potential complementary binding sites. (C) Nucleoplasma separation assay revealed the subcellular distribution of circ0093335. Positive controls of cytoplasm and nucleus were 18S and U6, respectively. (D) FISH assay to show the co‐localization of circ0093335 and miR‐338‐5p in Hep3B and HCCLM3, and DAPI was used for nucleus staining, scale bar, 50 μm. (E) miR‐338‐5p expression following circ0093335 overexpression in Hep3B and HCCLM3 cells. (F) RIP and qRT‐PCR were used to measure the relative enrichment of circ0093335 in Hep3B‐circ0093335 cells. (G) The luciferase activities were detected after miR‐338‐5p, miR‐NC, circ0093335‐WT, or circ0093335‐MUT transfection in HEK293 cells. (H) The relative expression of circ0093335 was measured after miR‐338‐5p or miR‐NC transfection. (I) CCK8 cell proliferation tests were carried out after miR‐338‐5p or miR‐NC transfection. (J, K) Transwell aggression experiments were conducted after transfections of the miR‐338‐5p mimic or miR‐NC, scale bar, 50 μm. *p < 0.05, **p < 0.01, and ***p < 0.001.

Recently, growing evidence has shown that miRNAs can modulate the actions of other ncRNAs. The probable targets of circ0093335 were predicted using a bioinformatics method to better understand the underlying mechanism (Circinteractome: https://circinteractome.nia.nih.gov/) (Figure 4A). The results showed that miR‐338‐5p has a tumour‐suppressive effect on gliomas and oesophageal squamous cell carcinomas (ESCCs). MiR‐338‐5p is a probable target of circ0093335, and its potential binding site was predicted (Figure 4B). Then, circ0093335 WT and MUT luciferase plasmids were synthesized, which had the binding site for the putative miR‐338‐5p. A nuclear and cytoplasmic extraction test was performed, which showed that circ0093335 was primarily localized in the cell cytoplasm (Figure 4C). We also conducted a FISH experiment to demonstrate that the majority of circ0093335 (red) and miR‐338‐5p (green) are co‐located in the cytoplasm of HCC cells (Figure 4D). MiR‐338‐5p expression was downregulated in OE‐circ0093335 cells (Figure 4E). Moreover, circ0093335 was enriched by Ago2 over IgG antibody, suggesting that it may serve as a miR‐338‐5p ‘sponge’, based on the results of RIP experiments (Figure 4F). A dual‐luciferase reporter test also consistently showed that miR‐338‐5p could reduce the luciferase enzyme activity in the circ0093335‐WT as compared with the circ0003998‐MUT (Figure 4G). These findings indicated that circ0093335 directly targeted miR‐338‐5p.

Additionally, the rescue studies suggested that transfection of the miR‐338‐5p mimic lowered circ0093335 expression level (Figure 4H). MiR‐338‐5p abolished the cancer‐promoting effects of circ0093335 overexpression (Figure 4I,J,K). These findings suggested that circ0093335 promotes tumour growth by sponging miR‐338‐5p in HCC.

## 4 | DISCUSSION

CircRNAs are a new family of ncRNAs that lack a 5′‐end cap or 3′‐end poly (A) tail, are widely found in plant and animal cells in a closed loop structure.^9^ They can exist independently of proteins, are not affected by exonucleases, and can modulate gene expression due to their specific structure.^26^ Recently, growing research has demonstrated the regulatory role of circRNAs in the onset and advancement of numerous malignancies, including HCC, cell cycle, cell proliferation, apoptosis and metastasis.^27^ Li et al. reported that the oncogenic circRNA circ0000098 facilitated HCC advancement via the miR‐383/MCUR1 axis.^16^ Li et al. demonstrated that the miR‐148a/STX3‐PTEN axis was the mechanism through which circMRPS35 triggered its carcinogenic effect in HCC.^28^ Liu et al. showed that the impact of upregulated circTOLLIP on the miR‐516a‐5p/PBX3/EMT axis, which enhances HCC metastasis.^15^ Liu et al. showed that downregulated circ‐ZEB1 decreased HCC advancement by controlling the miR‐199a‐3p/PIK3CA pathway.^29^ Ni et al. demonstrated that circ 0011385 knockdown decreased HCC growth via the miR‐361‐3p/STC2 axis.^30^ Zhang et al. showed that circC16orf62 accelerated HCC development via altering the miR‐138‐5p/PTK2/AKT axis.^31^ Hu et al. demonstrated that circASAP1 regulated the miR‐326/miR‐532‐5p axis to promote HCC advancement.^32^

Since, HCC is a multistage and multifactorial hereditary cancer. Thus, it is still worthwhile to investigate how circRNAs regulate HCC. This study revealed that circ0093335 expression had significantly higher levels in HCC cells and tumour specimens. According to the patient's clinical data assessment, high circ0093335 expression was linked with the TNM stage and MVI. Circ0093335‐overexpressed lentiviral vectors and NC lentiviral vectors were transfected into HCC cells, and cell function studies were conducted to explore the effect of circ0093335 on HCC development. These findings revealed that circ0093335 increased HCC cell proliferation and aggressive abilities in addition to mouse tumour growth.

CircRNAs are mostly found in the cytoplasm and interact with miRNAs, binding to targeted miRNAs and inhibiting their post‐transcriptional regulatory functions.^10^, ^26^ To further investigate the mechanism of circ0093335 in HCC, we predicted the miRNAs that bind to circ0093335 via Circinteractome (https://circinteractome.nia.nih.gov/) and conducted literature studies on the candidate miRNAs. The human miR‐338‐5p gene is located on chromosome 17 and the mature miR‐338‐5p length is 22 nt. It was first found in colorectal cancer (CRC) research and then reported in HCC, ESCC, breast cancer, glioma and other types of cancer. It plays a variety of biological regulatory roles in various malignant tumours.^33^, ^34^, ^35^ Xu et al. reported that miR‐338‐5p expression was downregulated in hypoxic CRC cell lines and that miR‐338‐5p inhibition was closely related to hypoxia‐induced CRC resistance. Further mechanism study found that HIF‐1α mediates the inhibition of miR‐338‐5p, interferes with the regulation of miR‐338‐5p on the downstream direct target IL‐6, mediates the activation of STAT3/BCL2 signalling pathway and enhances CRC resistance to oxaliplatin.^36^ However, Yang et al. found that Hsa_circ_01370008 overexpression inhibited the expression of Ki67 and PCNA and the proliferation, invasion and EMT ability of CRC cells under normoxia. Mechanism research found that Hsa_circ_01370008 inhibits CRC progression by spongy absorption of miR‐338‐5p.^37^ In ESCC, the level of miR‐338‐5p in tumour tissue, serum samples and ESCC cells were significantly downregulated. MiR‐338‐5p can inhibit the migration and invasion of ESCC cells. Lin et al. showed that miR‐338‐5p plays an anti‐cancer role in ESCC and mediates the sensitivity to cisplatin by inhibiting FERMT2.^34^ Han et al. showed that miR‐338‐5p could regulate 5‐FU chemotherapy resistance and inhibit ESCC invasion by negatively regulating Id‐1.^38^ Sun et al. found that miR‐338‐5p expression was significantly down‐regulated in pancreatic cancer tissues. Overexpression of miR‐338‐5p inhibited EMT and metastasis of pancreatic cancer cells by targeting MAPK/ERK signal pathway.^39^ Yao et al. reported that circ_0030167 inhibits pancreatic cancer progression by sponging miR‐338‐5p targeting the Wnt8/β‐catenin axis.^40^

Previous studies have shown that miR‐338‐5p is dysregulated in HCC through miRNA array and database data analysis and has the potential to be used as a diagnostic marker.^41^, ^42^ However, its molecular mechanism is unclear and needs to be clarified. Furthermore, Zhao et al. demonstrated that the miR‐338‐5p expression level was notably downregulated in liver tumours clinical specimens, and miR‐338‐5p overexpression dramatically decreased HCC growth.^43^ Based on bioinformatic predictions and publications, we hypothesized that circ0093335 served as a pro‐cancer agent by interacting with miR‐338‐5p and performed a series of assays to validate this. We used the dual‐luciferase assay to demonstrate direct base complementary pairing between miR‐338‐5p and circ0093335. Additionally, we performed miRNA mimic rescue experiments to verify that circ0093335 functions as a tumour promoter by interacting with miR‐338‐5p. All findings supported the mechanical interaction between circ0093335 and miR‐338‐5p.

Currently, we cannot identify circ0093335 as a promising diagnostic biomarker and evaluate the prognosis of patients with HCC in terms of life expectancy and length of disease‐free survival due to our sample size limitation and missing data on return time visits. MiR‐338‐5p has been reported to regulate multidrug resistance in HCC.^43^ However, the absence of published reports on circ0093335 and chemotherapeutic drug therapy for HCC limits our discussion in this regard, which is critical for clinical treatment. Furthermore, the potential molecular mechanisms of circ0093335 remain to be further explored. Further multicentre, large‐sample studies are needed to assess the significance of circ0093335 in terms of HCC diagnosis and prognosis. Additionally, further research is needed to determine the molecular mechanism of circ0093335 and how it affects the effectiveness of medication in HCC. Further research on circ0093335 as a new marker in HCC management is hoped to be conducted.

## 5 | CONCLUSIONS

Circ0093335 expression was upregulated in HCC, and circ0093335 ectopic expression in HCC cells promoted cell proliferation, aggressive abilities and mouse tumour growth. The results showed that circ0093335 accelerated HCC progression by acting as a miR‐338‐5p ‘sponge’ and may be a candidate target for HCC therapy.

## AUTHOR CONTRIBUTIONS

**Xiangyu Qin:** Data curation (equal); methodology (equal); writing – original draft (equal); writing – review and editing (equal). **Lingyu Zhou:** Conceptualization (equal); writing – review and editing (equal). **Yaojie Shen:** Data curation (supporting); methodology (supporting); writing – review and editing (supporting). **Yuwei Gu:** Data curation (supporting); writing – review and editing (supporting). **Jia Tang:** Data curation (supporting); writing – review and editing (supporting). **Junwei Qian:** Data curation (supporting); writing – review and editing (supporting). **An Cui:** Data curation (supporting); writing – review and editing (supporting). **Mingquan Chen:** Conceptualization (supporting); funding acquisition (supporting); writing – review and editing (supporting).

## CONFLICT OF INTEREST STATEMENT

All authors state that no commercial relationships that might be considered potential interest conflicts existed during the study.

## Data Availability

The data that support the findings of this study are available from the corresponding author upon reasonable request.
